# RNA Editing—Systemic Relevance and Clue to Disease Mechanisms?

**DOI:** 10.3389/fnmol.2016.00124

**Published:** 2016-11-23

**Authors:** Jochen C. Meier, Svenja Kankowski, Heinz Krestel, Florian Hetsch

**Affiliations:** ^1^Cell Physiology, Technische Universität BraunschweigBraunschweig, Germany; ^2^Neurology, Universitätsspital und Universität BernBern, Switzerland

**Keywords:** RNA editing, epilepsy, cancer, mental disorders, glycine receptor, glutamate receptor, potassium channels, serotonin

## Abstract

Recent advances in sequencing technologies led to the identification of a plethora of different genes and several hundreds of amino acid recoding edited positions. Changes in editing rates of some of these positions were associated with diseases such as atherosclerosis, myopathy, epilepsy, major depression disorder, schizophrenia and other mental disorders as well as cancer and brain tumors. This review article summarizes our current knowledge on that front and presents glycine receptor C-to-U RNA editing as a first example of disease-associated increased RNA editing that includes assessment of disease mechanisms of the corresponding gene product in an animal model.

## Introduction

The conversion of genetic information from the DNA to the protein level includes the transcription of a given gene into RNA and its subsequent translation. Protein expression is tightly regulated at each step, and depends on the presence and activity of a bulk of proteins, which in most cases are themselves regulated by further proteins, hormones, metabolites or other modulators. Thus, any cell may generate nearly infinite expression profiles being able e.g., to differentiate in a tissue-dependent manner during development or to adapt to changes in environmental conditions. The variability of a genome is further increased by posttranscriptional modifications that change the genetic information during expression and lead to alternative variants of a given protein. Posttranscriptional modifications may occur at each step of gene expression and are carried out by evolutionarily conserved mechanisms. Commonly known posttranscriptional modifications are the splicing events in the nucleus, RNA editing and the specific insertion of a selenocysteine instead of a cysteine during translation. This review article focuses on RNA editing of nuclear transcripts in higher eukaryotes, its mechanisms, systemic relevance and association with development and disease.

## RNA Editing—Enzymes and Mechanisms

RNA editing was described for the first time in *Trypanosoma* where the mRNA of the mitochondrial gene coding for cox-2 was found to differ from its expected sequence in terms of four missing uridines at the 5′ end in comparison to the respective DNA sequence (Benne et al., 1986). Subsequently to this intriguing discovery, it became clear that this “RNA editing” event occurs much more frequently, and that about 60% of the genomic information in mitochondria of *Trypanosoma* is edited by insertion or deletion of uridines. Nuclear mRNA of higher eukaryotes was also found to be edited in a reproducible fashion. However, unlike *Trypanosoma*, editing of nuclear mRNA in higher eukaryotes is rather a consequence of amination or deamination of purines and pyrimidines than a consequence of nucleotide insertion or deletion. The prevalent type of RNA editing in higher eukaryotes results from two types of hydrolytic enzymatic deamination reactions. As discussed below in more detail, deamination of cytidine leads to the conversion to uridine (C-to-U), which is dependent on the APOBEC (“apolipoprotein B mRNA editing enzyme, catalytic polypeptide-like”) gene family and auxiliary proteins. Enzymatic deamination of adenosine yields inosine (A-to-I) and is dependent on the ADAR (“adenosine deaminase acting on RNA”) enzyme gene family. U-to-C and G-to-A RNA editing may also occur due to amination of the respective bases (Sharma et al., 1994; Grohmann et al., 2010; Niavarani et al., 2015; Knie et al., 2016).

## The ADAR Gene Family

**Figure 1:**
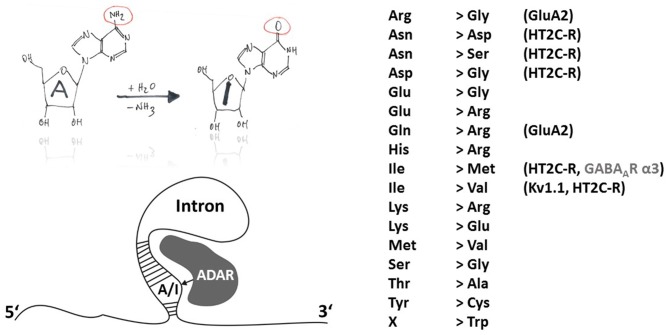
**Principles of A-to-I RNA editing.** The hand-drawing top left provides a schematic illustration of the deamination of adenosine to inosine (A-to-I). In contrast to APOBEC-dependent C-to-U RNA editing, ADARs do not need auxiliary proteins for deamination but rely on secondary mRNA structure motifs within introns (bottom-left). A-to-I RNA editing can lead to indicated (right-hand) amino acid recoding events. Selected edited products and their possible involvement in disease are discussed in this review article.

Members of the ADAR family catalyze the deamination of adenosine to inosine except in protozoa, yeast and plants (Jin et al., 2009). Inosin is interpreted as guanosine (G) during mRNA translation. ADAR principle function and sites of action are shown in Figure 1. ADAR enzymes are highly conserved among invertebrates and vertebrates. Three different *ADAR* genes (*ADAR1–3*) were identified in the mammalian genome with *ADAR1* being the first one (O’Connell and Keller, 1994; Kim et al., 1994a). During evolution, the ADAR family putatively arose from adenosine deaminases acting on tRNA (ADAT), which are conserved from yeast to man and also have a bacterial ortholog (TadA). Interestingly, the evolutionary predecessors of adenosine deaminases (ADAT and ADAR) are cytidine and not adenosine deaminases acting on mononucleotides, classifying ADARs, like APOBECs, into the cytidine deaminase family. This viewpoint is corroborated by comparison of the X-ray structures of the ADAR and APOBEC catalytic domains (for mechanisms of APOBEC-dependent RNA editing see below). The deamination motif of human ADAR2 bears two α-helices (α2 and α5) and four ß-strands that form a structure similar to the core of cytidine deaminase catalytic domains (Macbeth et al., 2005). Another similarity in the structural implications of APOBEC and ADAR is Zn^2+^ complexation at the active site of ADAR that is mediated by the histidine and glutamate residues of an HAE motif (aa 394–396 in human ADAR2) and the more distally located cysteine residues C451 and C516 (human ADAR2). Unlike APOBEC, members of the ADAR family are able to catalyze deamination without the aid of additional auxiliary proteins (Nishikura, 2010). This independent mode of action is perhaps intrinsic to several RNA binding domains located in the N-terminal part of the ADAR proteins. Thus, investigation of deamination mechanisms and the search for *in vivo* substrates may be more straightforward than in the case of the APOBEC enzymes. Catalytic activity has only been shown for the ubiquitously expressed ADAR1 and ADAR2 (Kim et al., 1994b; Higuchi et al., 2000) but not for brain specific ADAR3 which may be a regulatory component of the A-to-I editing machinery as it binds single and double stranded RNA and was shown to inhibit *in vitro* the activities of RNA editing enzymes of the ADAR gene family (Chen et al., 2000; Nishikura, 2010). Though most of the A-to-I RNA editing sites were attributed to non-coding genome regions such as *Alu* repeats or L1 LINE (Neeman et al., 2006), an increasing number of A-to-I RNA editing sites in protein coding regions is identified using advanced sequencing techniques (Levanon et al., 2005; Li et al., 2009; see http://www.rnaedit.com). Furthermore, there are some important indications that ADAR-mediated RNA editing modulates the efficiency of RNAi pathways including the generation of microRNAs (miRNAs) and the processing of small interfering RNAs (siRNAs) (Nishikura, 2006). However, 17 different amino acid recoding events can occur due to A-to-I changes within codons (Figure 1).

RNA and DNA binding of ADAR1 have been attributed to Z-DNA binding domains, which might confer target site specificity of editing of viral RNA templates (Brown et al., 2000) and, thus, play a virus selective role in the host response to infection (George and Samuel, 2011). When ADAR1 or ADAR2 is incubated with double-stranded RNA (dsRNA; >20 bp), about 50% of the adenosine residues will be edited in a promiscuous fashion (Bass and Weintraub, 1987; Nishikura et al., 1991). However, dsRNA molecules with imperfectly matched base pairs lead to a more selective action of ADAR, indicating that the secondary structure of the RNA substrate is crucial for site specificity of an ADAR-mediated editing event (Figure 1). Thus, cellular editing by ADAR occurs preferentially in double stranded RNA with mismatches, bulges and internal loops, suggesting that target site selectivity is based on conformation rather than on a particular sequence (Bass, 1997). The type of mismatch greatly influences A-to-I editing efficiency, with A:C being preferred over A:A, A:G or A:U (Wong et al., 2001). Nonetheless, in some cases A-to-I RNA editing does not occur although the target position seems to be in the right context (Lehmann and Bass, 2000). Actually, epigenetic regulation can contribute to ADAR-dependent A-to-I RNA editing, as was recently revealed by investigation of the cellular epitranscriptome and identification of methyl-6-adenosine RNA modification (Saletore et al., 2013), which blocks ADAR-dependent RNA editing (Véliz et al., 2003) and may be a ground-breaking discovery for personalized medical care (see below for a discussion of A-to-I RNA editing-dependent mechanisms in disease). Another possibly promising pharmaceutical approach may consist in targeting RNA splicing. In fact, ADAR2 activity can lead to creation of new RNA splice sites in ADAR2-coding mRNA and lead to alternative ADAR splice variants with altered RNA editing capacity (Rueter et al., 1999).

## ADAR-Dependent RNA Editing in Development

Already 30 years ago, it was postulated that A-to-I RNA editing may play an important role in development (Bass and Weintraub, 1987). Indeed, recently, down-regulation of α3-GABA(A)R expression was shown to result from A-to-I RNA editing by ADAR1 or ADAR2 at the I/M site (AUA-to-AUG coding for isoleucine [I] and methionine [M], respectively, Figure 1), which impairs GABA(A)R α3 surface expression, suggesting that by trafficking control of α3-containing receptors RNA editing may facilitate the switch of subunit compositions during development and affect the subcellular distribution of α subunits in the adult brain (Ohlson et al., 2007; Daniel et al., 2011). Switching subunit compositions may accelerate GABAergic synaptic response kinetics and enhance spike-timing precision at more mature developmental states, as GABA(A)R α3 expression is developmentally downregulated and GABA(A)R α1-coding mRNA cannot be edited at the I/M position (because AUU encodes isoleucine in mouse, rat and human; Lavoie et al., 1997; Jüttner et al., 2001).

Due to the advances in sequencing techniques a recent study could survey changes in the global landscape of A-to-I RNA editing in human brain tissues and revealed many more gene products that undergo developmental and disease-specific changes in gene expression. Thereby a spatiotemporal atlas of RNA editing was created that revealed a dynamic profile of RNA editing (Hwang et al., 2016). This pioneering study revealed three patterns of uniquely regulated RNA editing sites during cortical development from fetal to old age comprising stably high, stably low, and increasing editing at given sites. The increasing pattern of A-to-I RNA editing included sites in vesicle or organelle membrane-related genes and glutamate signaling pathways. In two selected disorders, namely spinal cord injury and glioblastoma, perturbed A-to-I RNA editing could be demonstrated, as discussed below in more detail.

## ADAR-Dependent RNA Editing in Disease

The pioneer studies by Peter H. Seeburg revealed a critical position that, amongst others, determines the calcium permeability of the AMPA-type ionotropic glutamate receptor channel and is situated in the hairpin loop between transmembrane domains 1 and 3 (Seeburg et al., 2001). In this case, A-to-I RNA editing at the Q/R site (CAG-to-CGG) of the AMPA receptor subunit GluA2 occurs at a rate of virtually 100% (Figure 1). Genetically engineered mice that carry an editing resistant Q/R site in *Gria2*—with 70% GluA2 mRNA expression from wild-type and 75% editing vs. 100% in wild-type—develop severe epilepsy with generalized seizures and premature postnatal lethality (Brusa et al., 1995). A conditional mouse model with deficient GluA2 Q/R editing only in forebrain had its seizure origin in the hippocampus (Krestel et al., 2004). To test the hypothesis whether editing deficiency at the GluA2 Q/R site contributes to human mesial temporal lobe epilepsy (TLE), hippocampi from patients who had undergone surgery for pharmacotherapy-resistant epilepsy were analyzed. It turned out that: (i) the GluA2 Q/R site editing belongs to the stably high editing pattern with 100% editing in all patients analyzed aged from 5 years to 57 years at the time of surgery; (ii) epilepsy could not perturb this stably high pattern; and (iii) epilepsy in mice due to deficient GluA2 Q/R site editing probably was an artifact of genetic engineering (Krestel et al., 2013).

**Figure 2:**
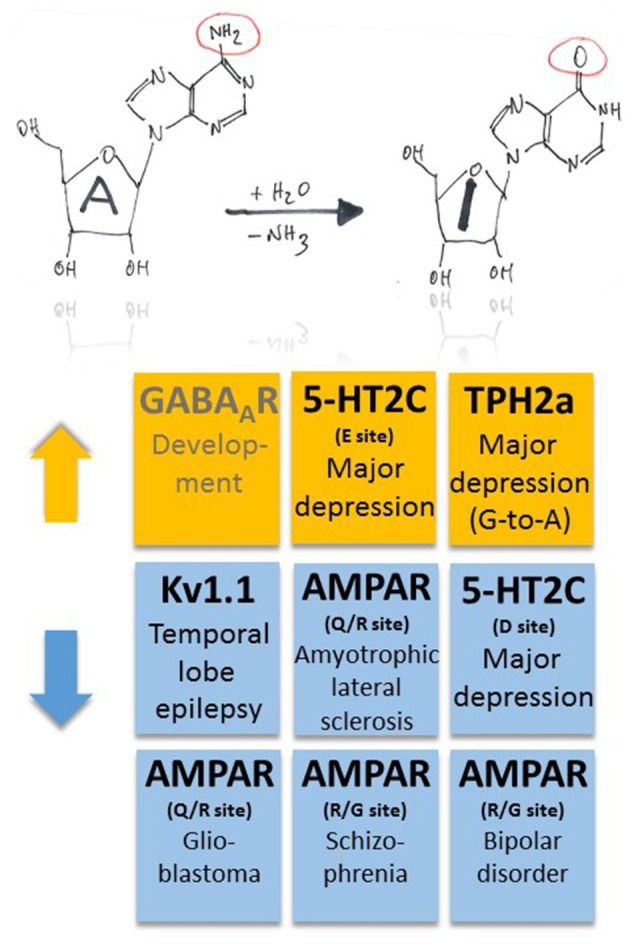
**Pathophysiological implications of altered ADAR-dependent RNA editing in disease.** Both up- and down-regulation of A-to-I RNA editing (indicated by orange and blue colors, respectively) were associated with several, different diseases. Note that up-regulation of GABA_A_R α3 RNA editing makes an exception as it plays a role in development (indicated by gray text color).

In patients with amyotrophic lateral sclerosis, decreased RNA editing at the Q/R site was found to occur in motor neurons (Kwak and Kawahara, 2005; Figure 2). In fact, ADAR2 gene targeting in motor neurons provokes a decline in motor function and the phenotype can be reverted if the mice express RNA edited GluA2 (Higuchi et al., 2000; Hideyama et al., 2010). Reduced A-to-I RNA editing at the Q/R site of GluA2 was furthermore shown to be associated with glioblastoma (Maas et al., 2001; Figure 2). However, in spinal cord injury and glioblastoma, the developmentally increasing pattern of A-to-I RNA editing of many gene transcripts seems to be disrupted (Hwang et al., 2016), raising the question about underlying mechanisms of these diseases (Fu et al., 2016), including investigation of “master” genes that regulate function of down-stream genes and thus govern signaling cascades involved in disease. Regarding GluA2 under-editing, which was more pronounced in glioblastoma compared to neighboring non-tumor tissue (Hwang et al., 2016), and taking into account that brain tumor cells can release glutamate by transferrin-mediated iron accumulation (Chirasani et al., 2009), reduced RNA editing and increased calcium signaling through GluA2 may contribute to aggressiveness of tumor growth and expansion. Regarding spinal cord injury in a mouse model (Chen et al., 2013), decreased RNA editing of GluA2 in the epicenter of the injured site actually points to a disease-promoting mechanism, as cellular calcium overload should promote neurodegeneration.

Moreover, changes in ADAR-mediated A-to-I RNA editing were associated with several other types of cancer, including breast cancer, neuroblastoma and hepatocellular carcinoma; for a review see Fu et al. (2016), suggesting that some common master gene products may be involved in the pathogenesis of these diverse diseases. Finally, in human postmortem brains of patients with schizophrenia and bipolar disorder ADAR2 expression tended to be decreased, and decreased ADAR2 expression was significantly correlated with decreased editing of the R/G sites of AMPA receptors (Kubota-Sakashita et al., 2014; Figures 1, 2). All these examples point to a crucial role of AMPA-type glutamate receptor RNA editing in many disease conditions, but the underlying mechanisms still remain obscured and need to be addressed. Although RNA splicing of ADAR2 may provide correlative mechanistic insights into the interrelation between enzyme function and RNA editing (Fu et al., 2016), identification of a general downstream mechanism of disease manifestation, e.g., changes in calcium signaling, would eventually reveal promising strategies to tackle these diseases early in their pathomechanisms.

Serotonergic neurotransmission was shown to be influenced by RNA editing of serotonin receptors (5-HT2C-R; Gurevich et al., 2002a; Figures 1, 2). A-to-I RNA editing of 5-HT2C-R at up to five positions decreases the apparent receptor affinity to serotonin, with codon 158 (AAU-to-AGU [C site] or –GAU [E site] or –GGU [C and E sites]) in the intracellular loop between transmembrane domains 3 and 4 producing the strongest effects (up to a 20-fold decrease; Fitzgerald et al., 1999). Editing of 5-HT2C-R thus seems to be a versatile tool for neurons to dynamically adjust receptor response properties to alterations in serotonin levels. This is an economic way of regulation as it bypasses the need for supplementary genes (or exons that could be alternatively spliced) and associated regulation of gene expression. However, the mechanism may also fail as a complex pattern of altered RNA editing in depressed suicide victims was suggested to exacerbate the effects of low serotonin (Gurevich et al., 2002b; Figure 2). Furthermore, G-to-A RNA editing occurs in transcripts coding for tryptophan hydroxylase 2 (TPH2a) that controls brain serotonin synthesis (Grohmann et al., 2010). In this case, G-to-A editing leads to amino acid substitution R441H that decreases TPH2a enzyme activity and, thus, may also contribute to major depression disorder (Grohmann et al., 2010; Figure 2). In fact, coincident changes in RNA editing of both 5-HT2C-R and TPH2 may result in a cumulative decrease in 5-HT signaling, constituting a worst-case scenario for the patients.

Potassium channels also undergo A-to-I RNA editing which substitutes valine for isoleucine at position 400 (I400V) within the transmembrane domain 6 of human Kv1.1 (Bhalla et al., 2004). The resulting accelerated recovery from inactivation and increase in K^+^ outward current upon membrane depolarization (Bhalla et al., 2004) can stabilize repolarization through Kv1.1 potassium channels and thus control neuronal excitability. The Kv1.1 I/V site belongs to the “increasing pattern” observed in human brain development (Hwang et al., 2016), as mentioned above. On the other side, a decrease of RNA editing may translate into destabilized repolarization and may contribute to the maintenance of neuronal hyperexcitability. Remarkable in this context is the observation that the RNA editing rate decreases with epilepsy duration in the removed hippocampus of patients who underwent surgery for intractable mesial TLE with hippocampal sclerosis but not with other clinical parameters (Figure 2). A specific association of the decrease with either the epileptic process itself or its antiepileptic medication history was suggested (Krestel et al., 2013).

## The APOBEC Gene Family

The successful cloning of *APOBEC-1*, the first identified member of the *APOBEC* gene family of cytidine deaminases (Navaratnam et al., 1993; Teng et al., 1993), signified an important hallmark in research of the molecular components of the mammalian C-to-U editosome. APOBEC is a family of evolutionarily conserved proteins. As of today, 10 additional cytidine deaminases homologous to APOBEC-1 and thus contributing to the *APOBEC* gene family were identified (APOBEC-1, -2, -3A, -3B, -3C, -3D, 3E, -3F, -3G, -3H, -4; activation-induced cytidine deaminase (AID); Bransteitter et al., 2009). While members of the APOBEC enzyme family are responsible for editing of pre-mRNA, single stranded DNA (ssDNA) and genomic DNA, AID seems to mostly edit ssDNA and contribute to the adaptive immune response by introducing dC-to-dU mutations into the VDJ region of the immunoglobulin gene (Conticello, 2008). APOBEC-1 is the best characterized member of the *APOBEC* gene family. Thanks to the pioneer work of Harold C. Smith, the role of APOBEC-1 and auxiliary proteins in pre-mRNA editing of apolipoprotein B (ApoB) was elucidated in great detail (Backus and Smith, 1992; Schock et al., 1996; Dance et al., 2002; Smith, 2007). The role of APOBEC-2 was discovered more recently. APOBEC-2 was shown to be involved in C-to-U RNA editing of eukaryotic translation initiation factor 4 gamma 2 as well as phosphatase and tensin homolog (PTEN) genes and to be associated with tumorigenesis (Okuyama et al., 2012). In addition, APOBEC-2 plays a crucial role in muscle development (Sato et al., 2010) and in the TGFß-mediated manifestation of internal organ left-right asymmetry during development (Vonica et al., 2011). APOBEC-3A-H can inhibit the propagation of HIV and human papillomavirus (HPV) by editing the viral double-stranded cDNA intermediates that serve as template for the expression of viral proteins (Conticello, 2008; McDougall et al., 2011). APOBEC-3B may preferentially edit genomic DNA and is implicated in cancer as its expression correlates with increased DNA damage and thus represents an enzymatic source of mutation in breast cancer (Burns et al., 2013). APOBEC-3G edits ssDNA under certain conditions (McDougall et al., 2011). By deaminating C to U and thus inserting mutations, APOBEC-3G might protect the mammalian genome against the spread of retroviruses. Supportive evidence comes from the upregulated expression of APOBEC-3G in inflammatory skin disorders such as *Lichen planus*. This disease is believed to be associated with activation of quiescent human endogenous retroviruses, and upregulation of APOBEC-3G is perceived as an endogenous defense mechanism (Nogueira et al., 2015). APOBEC-4 was found by *in silico* methods (Rogozin et al., 2005) and appears to influence HIV-1 expression (Marino et al., 2016).

**Figure 3:**
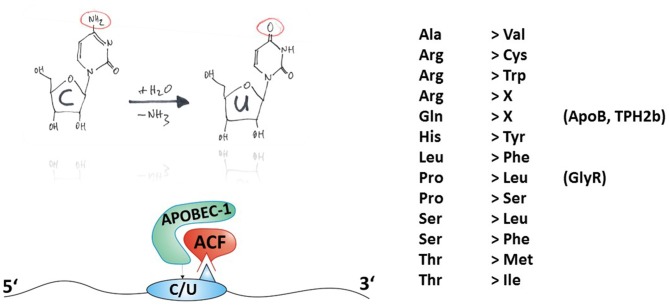
**Principles of C-to-U RNA editing.** The hand-drawing top left provides a schematic illustration of the deamination of cytidine to uridine (C-to-U). In contrast to ADAR-dependent A-to-I RNA editing, APOBEC needs auxiliary proteins for deamination as detailed in Table 1 and exemplified in the bottom-left panel. Thus, the mature mRNA can be RNA-edited. C-to-U RNA editing can lead to indicated (right-hand) amino acid recoding events. Some selected gene products associated with disease (in brackets) and their possible involvement in disease are discussed in this review article.

A common feature of all members of the APOBEC family is an N-terminal catalytic domain comprising the sequence HXEX_27/28_PCXXC (Jarmuz et al., 2002) that coordinates a Zn^2+^ ion within the active center of the enzymes. The tertiary structure of APOBEC catalytic domains, derived from X-ray and NMR studies on APOBEC-2 (Prochnow et al., 2007) and APOBEC-3G (CD2, a C-terminal additional catalytic domain only present in some APOBEC isoforms; Chen et al., 2008; Holden et al., 2008), is composed of a five ß-strands containing ß-sheet that is surrounded by six α-helices. In the case of full-length APOBEC-2, homo-tetramerization has been shown to prevent the active site of the catalytic domains to be accessible to nucleic acids (Prochnow et al., 2007), counteracting the catalytic activity of the enzyme. This self-inhibitory action of APOBEC-2 might explain why demonstration of *in vivo* deamination activity of this isoform is rather challenging. However, the C-to-U RNA editing mediated by APOBEC-1 depends on many auxiliary proteins (Blanc and Davidson, 2010; Table 1 and Figure 3). Although APOBEC-1 itself can accommodate RNA substrates involving zinc-finger domains and, additionally, two critical phenylalanines (Anant et al., 1995; Navaratnam et al., 1995), APOBEC-1 complementation factor (ACF) is essential for editing activity (Schock et al., 1996; Mehta et al., 2000). ACF contains RNA recognition motifs (RRM) that direct APOBEC-1 to the target sites (Lellek et al., 2000; Mehta et al., 2000; Henderson et al., 2001). These RRM domains bind to the mooring sequence UGAUCAGUAUA located downstream of the edited position and thus confer target specificity (Smith, 2007), while the C-terminal auxiliary domain of ACF is required for interaction with APOBEC-1 (Mehta and Driscoll, 2002). Furthermore, the RNA-binding protein CUGBP2 is part of the APOBEC-1 holoenzyme and specifically targets AU-rich elements immediately upstream of the edited position (Anant et al., 2001). Many other factors have also been shown to modulate ApoB RNA editing, although in these cases the mechanisms are less well understood (Table 1). *APOBEC-1* knockout mice do not edit ApoB mRNA (Hirano et al., 1996; Nakamuta et al., 1996), even though these mice express other members of the *APOBEC* gene family, indicating the intriguing specificity of this editing process. APOBEC-1 and ACF are present in both cytoplasm and nucleus but within the progress of protein expression, ApoB mRNA editing appears to be not cytosolic but intra-nuclear and post-transcriptional (Lau et al., 1991; Blanc and Davidson, 2010). A detailed review of the current knowledge about ApoB mRNA editing by APOBEC-1 was recently published by Harold C. Smith (Prohaska et al., 2014).

**Table 1:** **APOBEC-1 auxiliary proteins**.

Name	Function	Effect on C-to-U RNA editing	Reference
ACF	RNA binding (mooring sequence UGAUCAGU-AUA) and interaction with APOBEC-1	↑	Mehta and Driscoll (2002)
			Sowden et al. (2004)
CUGBP2	RNA binding (AU-rich elements) and ACF interaction	↑	Anant et al. (2001)
GRY-RBP	RNA binding and interaction with ACF and APOBEC-1	↓	Blanc et al. (2001)
hnRNP C1	RNA binding and interaction with APOBEC-1	↓	Greeve et al. (1998)
ABBP-2	Interaction with APOBEC-1	↑	Lau et al. (2001)
BAG-4	Interaction with APOBEC-1 and re-routing to the perinucleolar compartment	↓	Lau and Chan (2003)
AUX240	Editosome assembly	↑	Schock et al. (1996)

C-to-U RNA editing can lead to 13 different amino acid recoding events, with two of them leading to a STOP codon, as shown in Figure 3. In the following section, examples of amino acid recoding C-to-U RNA editing associated with disease will be presented.

## APOBEC-Dependent RNA Editing in Disease

**Figure 4:**
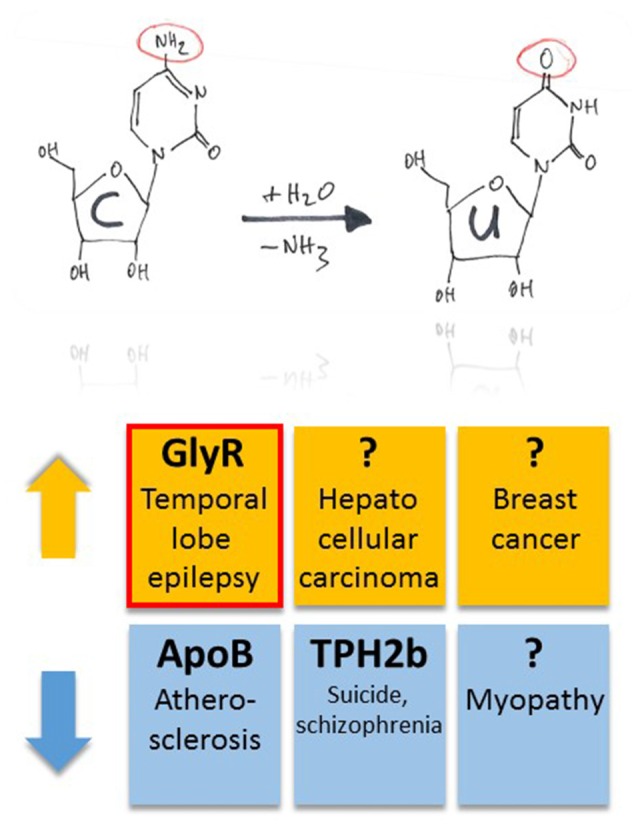
**Pathophysiological implications of altered APOBEC-dependent RNA editing in disease.** Both up- and down-regulation of C-to-U RNA editing (indicated by orange and blue colors, respectively) were associated with several, different diseases. However, compared to diseases that have been associated with altered A-to-I RNA editing, this list is rather short. In particular, in half of the cases, the question marks indicate global changes in C-to-U RNA editing due to changes in enzyme expression without identification of specific gene products that were associated with up- or down-regulation of the APOBEC enzymes in animal models or tissue samples of patients. The red framed example is discussed in more detail in this review article.

The mRNA coding for ApoB is C-to-U RNA edited (Boström et al., 1990). By regulating low and high density lipoprotein metabolism, RNA editing and the resulting biosynthesis of truncated ApoB48 protein is a critical regulator of plasma cholesterol content (Nakamuta et al., 1996), and dysregulation of APOBEC-1-dependent ApoB48 expression results in hypercholesterolemia and atherosclerosis (Fu et al., 2004; Figure 4).

As mentioned above, G-to-A editing leads to amino acid substitution R441H that decreases TPH2a enzyme activity (Grohmann et al., 2010). However, the alternatively spliced TPH2b enzyme can be C-to-U RNA-edited within the splice insert exon 3b, leading to c.385C>T and a truncated protein variant due to Q129X substitution (Grohmann et al., 2010). Editing at this position of TPH2b transcripts of suicides and patients with schizophrenia decreased substantially by 50% and 30%, respectively (Grohmann et al., 2010; Figure 4).

Changes in C-to-U RNA editing were also associated with several other diseases. In particular, APOBEC-2 deficient mice showed a markedly increased ratio of slow to fast fibers in soleus muscle and exhibited a reduction in body mass from birth onwards, with elderly mutant animals revealing clear histological evidence of a mild myopathy (Sato et al., 2010; Figure 4). Increased expression of APOBEC-2 in the liver resulted in significantly high frequencies of nucleotide alterations in the transcripts of eukaryotic translation initiation factor 4 gamma 2 as well as PTEN and was accompanied by hepatocellular carcinoma in 10% of 72 weeks old animals as well as lung tumors in 35% of transgenic mice analyzed (Okuyama et al., 2012; Figure 4).

APOBEC-3B seems to be an enzymatic source of mutation in breast cancer (Burns et al., 2013). In particular, the DNA cytosine deaminase activity of APOBEC-3B was shown to be up-regulated in primary breast tumors and breast cancer cell lines (Figure 4), suggesting that APOBEC3B-catalyzed deamination provides a chronic source of DNA damage in breast cancers that could select TP53 inactivation and explain how some tumors evolve rapidly and manifest heterogeneity. (Burns et al., 2013). Surprisingly, in the case of *Wilms Tumor 1*, APOBEC-3A was associated with a novel form of G-to-A editing, perhaps opening a way to further investigations into the mechanisms of other potential mRNA changes and helping us to redefine the RNA editing paradigm in both health and disease (Niavarani et al., 2015).

Although APOBEC-4 did not show any deamination activity, it was shown to enhance the replication of HIV-1, suggesting a natural role in modulating host promoters or endogenous long terminal repeat (LTR) promoters rather than being a cytosine deaminase (Marino et al., 2016).

Again, the description of global changes in C-to-U RNA editing due to changes in APOBEC-2, APOBEC-3 or APOBEC-4 function is not sufficient and can just be a starting point for the investigation of the critical targets that govern disease progression in the various kinds of diseases described here.

**Figure 5:**
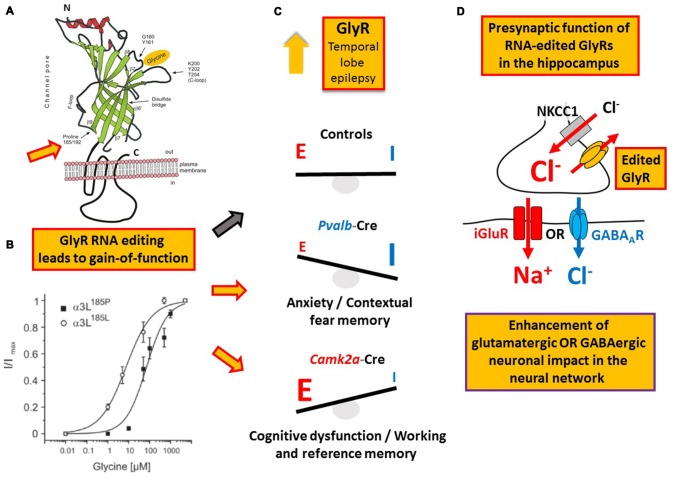
**Example of a RNA editing-dependent bedside-to-bench approach. (A)** GlyR C-to-U RNA editing leads to amino acid recoding in the ligand binding domain (P185L or P192L, indicated by the orange filled red framed arrow); modified from Legendre et al. (2009). **(B)** The amino acid recoding increases apparent GlyR affinity to glycine irrespectively of the subunit composition as discussed in the main text. Panel **(B)** is reproduced from Legendre et al. (2009) and shown here again for the purpose of clarity. **(C)** Increased RNA editing of GlyR-coding mRNA in patients with temporal lobe epilepsy (TLE) as documented in 2008 by Eichler and colleagues. In a corresponding mouse model of TLE RNA-edited GlyRs elicited different neuropsychiatric symptoms depending on the neuron type that expressed these receptor variants, compared to control animals without expression of RNA-edited GlyR and indicated by red framed yellow arrows and the black framed gray arrow, respectively, placed between **(B,C)**. **(D)** It is the presynaptic function of RNA-edited GlyRs that is responsible for increased neuronal impact on network function and the neuron type-specific neuropsychiatric symptoms described in the main text and shown in panel **(C)**.

Like GABA type A receptors the neurotransmitter receptors for glycine (GlyRs) belong to the ligand-gated ion channel gene superfamily and are glycine-gated chloride channels which were involved in TLE, inflammatory pain sensitization, autism spectrum disorder and glioblastoma (Harvey et al., 2004; Eichler et al., 2008; Förstera et al., 2014; Pilorge et al., 2016). GlyRs are C-to-U RNA edited although the mooring sequence recognized by ACF is not very well conserved (Meier et al., 2005). In this case, C-to-U RNA editing leads to a gain-of-function as the resulting amino acid substitutions P185L (in GlyR α1 and α3 subunits) and P192L (in GlyR α2) increase apparent agonist affinities of the neurotransmitter receptors (see Eichler et al., 2008; Legendre et al., 2009; and Figures 5A,B). Actually, C-to-U RNA editing of GlyRs was shown to be increased in the hippocampus of patients with pharmacoresistant TLE (Eichler et al., 2008; Figures 4, 5), suggesting that it plays a critical role in this disease. To test this hypothesis, a corresponding animal model was generated and allowed investigation of neuron type-specific mechanisms of RNA-edited GlyR action (Winkelmann et al., 2014). In sharp contrast to the still prevailing dogma that GlyRs are exclusively located at the postsynaptic site of synaptic signaling (Tyagarajan and Fritschy, 2014), we found that RNA-edited GlyRs are expressed at the presynaptic terminals of hippocampal neurons (Winkelmann et al., 2014). This is due to RNA splicing of the GlyR α3 subunit (Nikolic et al., 1998; Eichler et al., 2009) and substantiated for other GlyR subunits due to the absence of GlyR β protein in the hippocampus (Weltzien et al., 2012). Actually, other researchers also highlight the importance of presynaptic GlyRs (for examples see Lee et al., 2009; Kubota et al., 2010; Waseem and Fedorovich, 2010). However, even if GlyRs are expressed at both presynaptic and postsynaptic sites, a small number of presynaptic RNA-edited gain-of-function GlyRs even a single cluster of the non-RNA-edited GlyR α3L splice variant, which contains up to 200 receptor channels (Notelaers et al., 2012), will have a greater impact on the presynaptic membrane potential due to the much smaller volume and hence electrical capacity of this compartment compared to the somatodendritic compartment (Meier et al., 2014). Notably in this context, application of a low glycine concentration (10 μM) to hippocampal slice preparations enhanced the occurrence of epileptiform activity whereas a high glycine concentration (100 μM) attenuated recurrent epileptiform discharge (Chen et al., 2014). These divergent effects can be explained by preponderant functional impact of low glycine on presynaptic GlyRs expressed at glutamatergic terminals, resulting in facilitated glutamate release, and massive recruitment of somatodendritic GlyR activation by 100 μM glycine, resulting in tonic inhibition, respectively. Based on current evidence we believe that the presynaptic compartment is particularly vulnerable to maladaptive changes in neurotransmitter receptor signaling in disease (for a review see Meier et al., 2014). In the context of the rather low neuronal ambient glycine concentration in the hippocampus, presynaptic RNA-edited GlyRs were indeed shown to facilitate neurotransmitter release and contribute to gain-of-function of the affected neuron types, which elicited neuropsychiatric symptoms like cognitive dysfunction or persistence of contextual fear memory in our animal model. The different symptoms depended on the neuron type that expressed the RNA-edited GlyR variant, namely glutamatergic principle neurons (Camk2a-Cre) and parvalbumin-positive neurons (Pvalb-Cre), respectively (Winkelmann et al., 2014; Çalişkan et al., 2016; Figures 5C,D). As these symptoms of the genetically targeted mice are reminiscent of the disease symptomatology of TLE patients, we are currently investigating whether novel molecular and chemical tools (antagonists of RNA-edited GlyRs) are able to identify the neuron types with increased GlyR RNA editing in the hippocampus of patients with TLE and to counterbalance GlyR-dependent changes in neural network excitability. However, and most importantly, these studies demonstrate that it is not sufficient to study changes in RNA editing using bulk material because the same RNA-edited gene product (i.e., GlyR) can elicit completely different symptoms, depending on the neuron type that expresses the pathogenic RNA-edited protein variant.

## Conclusion and Perspective

RNA editing is an evolutionarily conserved process that has several advantages over gene mutation. Like alternative RNA splicing, the extent of RNA editing may be regulated, resulting in increased genomic variance. Furthermore, mRNA variability can be regulated whereas gene mutations are permanent. It seems as if there is reasonable evidence supporting altered RNA processing in a wide range of disease, including paroxysmal and neoplastic disorders of the brain, neuromuscular disease, as well as lung and liver disease and breast cancer. The examples discussed in this review article actually point to a critical role of deviation from normal RNA processing/editing in disease ontology and/or the epitranscriptomics of disease. However, there are several hundreds more amino acid recoding RNA editing events in many different gene products including gene regulatory transcripts (see http://www.rnaedit.com) that require investigation at the functional level; some of which may turn out to be the master regulatory targets of the editing machinery and disease-promoting cellular programs. Thus, we are just at begining of a new era of research that will ultimately need to identify functional changes of gene products due to amino acid recoding RNA editing and identify molecular key players that are masters and hence could signal upstream of, and regulate RNA processing machines. Furthermore, using animal models, in depth characterization of the functional consequences at both molecular and cellular levels are required to identify mechanisms that are responsible for the diverse phenotypes and disease symptomatology. The fact that there is an increasing number of patients with unknown cause of disease further underscores the need for characterization of causes and effects of epitranscriptional pathophysiological deviation from normal RNA processing, hopefully providing a future good starting point for the development of novel, genuine, therapeutic concepts.

## Author Contributions

All authors contributed to writing the manuscript.

## Conflict of Interest Statement

The authors declare that the research was conducted in the absence of any commercial or financial relationships that could be construed as a potential conflict of interest.
